# Hydrorrhœa

**Published:** 1854-09

**Authors:** 


### ECLECTIC DEPARTMENT,

### AND SPIRIT OF THE MEDICAL PERIODICAL PRESS.

---

*Hydrorrhœa.* By J. L. Twyman, M. D., of Amherst, Va.

On the 4th day of July, 1853, I was called to see Mrs. ——— who supposed herself to have been pregnant a month or more, and who had just had a watery flow from the vagina, represented to be, in quantity, a little less than a pint. A short time previous to the occurrence, she felt some uneasiness in the back and lower part of the abdomen, but was not sufficiently inconvenienced to cause her to go to bed, nor did she apprehend any unpleasant occurrence, till she found a sudden flow of water from the vagina. The principal part of the water was discharged in a quarter or half hour, but there was a slight dribbling till some time the next day. The fluid evacuated was represented was represented to be as thin as water, semi-transparent, and was found, upon examination, to have produced a faint, reddish stain upon the linen. About four weeks before that time she had a similar attack, and voided water in the same way, but not so much as at this time.

I saw her a few hours after the vaginal discharge; found her in bed, complaining of general weakness, pain in the back and lower part of the bowels; made no vaginal examination—supposed that nothing could be detected by it at this period of the pregnancy—gave her a quarter of a grain of sulphate of morphia, advised her to keep still in bed, to have the windows of her room hoisted, as the weather was warm, and to use cold acid drinks. After remaining in bed abot a week she was able to get up and go about as usual. This lady was about twenty years of age, fair skin, blue eyes and delicate frame, was married the December preceding, had menstruated somewhat irregularly till about the first of April, which was the last time, and afterwards suspected herself to be pregnant. She had no further return of the discharge after the 4th of July; soon had unequivocal symptoms of pregnancy, and on the 21st of January last was delivered of a healthy, well-formed female child of medium size. Both mother and child did well. As she lived out of the range of my practice, and returned home before her confinement, I can say nothing in reference to the character of her accouhement.

Estimating the term of utero-gestation in this case at nine calender months—the child being born on the 21st of January, 1854—the mother was impregnated on the 21st April preceding. At the time of the first vaginal evacuation, which was about the first of June, she had been pregnant forty days, and at the second, which took place on the 4th July, seventy-four days.

Different opinions have been entertained by different writers upon this subject, as to the influence which is exerted by these watery discharges, or their causes, upon the well being of the mother and the fœtus. The opinion prevails with many, not only with the populace, but among the profession, that any copious discharge from the womb, antecedent to the full term of utero-gestation, is necessarily followed, in a short time, by abortion or premature labor. Such however, is not the fact. Many cases are on record, in which the woman lost a large quantity of a serous fluid daily, and for a long time, without any injurious effect upon either the mother or child. Out of many cases which might be referred to, to establish the position that such discharges are borne for a long time, without any pernicious effect upon either the mother or fœtus, the following may be cited: "A woman, twenty-eight years of age, was seized in the fourth month of pregnancy, with a discharge of clear lymph from the vagina, so that she voided of this transparent fluid about two pounds daily. On the third day after the accession of the flux, she was attacked with fever, in consequence of which it sustained an inconsiderable diminution in its quantity, but was not suppressed. The fever was repressed by bleeding and the use of cinchona bark. The flux of lymph, however, continued during the whole of her pregnancy; but during the latter months, only in the quantity of about half a pound daily. About the eighth month, the patient fell into a violent passion which was followed by the accession of labor pains, and was delivered of a healthy living child soon afterwards." *Comment de Rebus in Scient. Nat. et Med. vol. iii. p.* 648: *Leipsic*, 1754. Quoted by Churchill—Diseases of Pregnancy and Childbed.

Here, then, is a case in which, from the fourth month of pregnancy to the eighth, when the child was born, there was a loss of fluid varying from a half pound to two pounds daily, without the destruction of either the mother or child. Some obstetricians, on the other hand, think such cases are attended with great danger. Dr. Davis, a distinguished obstetrical practitioner of London, says: "The escape in dribbling quantities, of an aqueous fluid, similar to liquor amnii, for many weeks or months before the accession of labor, is, in most cases, a dangerous, and often a fatal affection of the pregnant state."—*Obstetric Medicine.*

Churchill, in commenting upon this opinion of Dr. Davis, says, "it is at variance with other authorities, who do not generally consider it of so serious a character."—*Diseases of Pregnancy and Childbed.*

Cases of the character of those above described are quite rare, the writer never having seen but the one in more than twenty years' practice; but a *few* have been observed by many practitioners, among whom may be mentioned Heberden, Burns, Davis, Siecbold, Velpeau, Naegile, Churchill and others.

The pathology too of this affection is yet an unsettled question in medicine. By some it is considered a discharge of the amniotic liquor, which escapes either by transudation or by rupture; other look upon it as coming from the cavity of the chorion. Churchill says, "it is considered by some to be an excessive secretion from the glands of the cervix uteri," while he himself thinks "it not improbable that it may have its seat in the lining membrane of the vagina." M. Velpeau is of opinion, that in a large number of cases the source of the flow is in the cavity of the decidua, while M. Naegile of Heidelberg suggests that the fluid is secreted by the uterus itself, and this fluid, by gradually detaching the membranes from its inner surface, finds its way down into the vagina, whence it escapes.

Whatever may be the source of the flow in some cases, it is almost certain in others that it is derived from the amnois. The editor of Gooch's

Midwifery, at page 94, says: "A case has fallen within my knowledge, in which the membranes ruptured and the liquor amnii discharged at the fifth month of pregnancy; trifling portions of this fluid continued to be discharged at frequent intervals during ten subsequent weeks, at the end of which time a living child was born, which survived about ten day. There was no appearance of liquor amnii at the time of the delivery." Many other cases might be adduced corroborative of the same opinion. Dr. J. Young, of Chester, Pennsylvania, mentions a case wherein the membranes, as he thinks, were ruptured the first of April—the woman then being two months gone—and the water continued to flow moderately almost constantly till the last of October, when she gave birth to a living child. At the time of the birth there was no sudden gush of the waters, but the case was, in every respect, similar to an ordinary labor, in which the waters had been recently discharged.—*Am. Jour. Med. Sciences, N. S. vol. 23d.*

These two cases very much favor the idea that the discharge is from the cavity of the amnios; but it is not improbable that the source of the flow in other cases is from a different cavity or part of theuterine apparatus, inasmuch as, in some, attended by frequent, copious and long contined discharges from the cavity of the womb, when labor came on, the membranes protruded through the ostincæ in the usual way, finally burst, and gave vent to the ordinary quantity of liquor amnii. As there is, in all probability, no reparative process by which the torn edges of the amnios could ever again, after being once ruptured, become so united as to retain the liquor amnii, we can hardly resist the conclusion, in those cases in which quantities of water had been discharged long antecedent to the birth of the child, that the existence of a "protruding bag of waters" at the time of the birth, is proof pretty positive that the evacuation in preceding stages of utero-gestation was *not* from the cavity of the amnios.

If the discharge ever take place from the vagina, as suggested by Churchill, can it be upon the principle of endosmose of the serum of the blood? The experiments of Poiseville establish the fact that endosmotic action takes place through animal tissues from the serum of the blood, to Seidlitz water, and to solutions of sulphate of soda and common salt. Upon this physical law, he thinks, those substances act when taken internally. The endosmose, in this case, takes place through the capillary vessels of the intestine, from the serum of thd blood, to the saline solution introduced into the alimentary canal.—*Matteucci's Lectures on the Physical Phenomena of Living Beings, p.* 78.

Would it be more strange, in the event of some peculiar chemical action taking place in the secretions of the vagina, that endosmose of the serum of the blood might be established through the mucous tissue of that organ. This is thrown out as a suggestion, not as an opinion.

As cases of the disease noticed in this communication are seldom met with, I have thought it may be read with some interest by a portion, at least, of the junior members of the profession.—*Virginia Stethoscope.*

